# Cirrhosis of Liver in Patients With Dyskeratosis Congenita: A Report of Two Cases

**DOI:** 10.1002/ccr3.72044

**Published:** 2026-02-14

**Authors:** Bigyan Maharjan, Pradeep Neupane, Bikash Poudel, Ramesh Jha, Suraj Subedi, Jessica Baral, Abhishek Pandit, Shraddha Uprety, Mukesh Kumar Ranjan

**Affiliations:** ^1^ Department of Medical Gastroenterology Chitwan Medical College Bharatpur Chitwan Nepal; ^2^ Department of Dermatology, Venerology and Leprology Chitwan Medical College Bharatpur Chitwan Nepal

**Keywords:** bone marrow failure, cirrhosis, dyskeratosis congenita, esophageal varices, telomere biology disorders

## Abstract

Dyskeratosis congenita (DC) is a genetic disorder characterized by multisystem involvement. The most commonly affected systems are the mucocutaneous, bone marrow, and lungs. Though rare, chronic liver disease (CLD) has increasingly been reported to be associated with DC. The hepatic involvement can range from asymptomatic transaminasemia to end‐stage liver disease. In this series, we present two cases of adult male patients with DC and decompensated CLD.

## Key Clinical Message

Dyskeratosis congenita, a form of telomeropathy, should be considered in young patients with cryptogenic cirrhosis, especially when accompanied by cytopenias. Clinicians should actively look for the mucocutaneous triad, which includes reticular hyperpigmentation, nail dystrophy, and oral leukoplakia. Though DC is an incurable disease, early recognition enables timely surveillance and management of portal hypertension–related complications.

## Graphical abstract

Classical mucocuaneous triad of dyskeratosis congenita in a patient with decompensated cirrhosis.

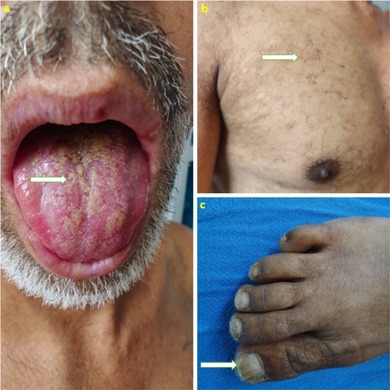

## 1 Introduction

Dyskeratosis congenita (DC) is a rare, inherited, multisystem disorder of telomere maintenance (telomeropathy). DC is an X‐linked disorder that can manifest with autosomal dominant or recessive inheritance patterns. It typically manifests during childhood or adolescence, characterized by a mucocutaneous triad comprising reticular skin hyperpigmentation, nail dystrophy, and oral leukoplakia [1]. Skin changes most commonly affect sun‐exposed areas such as the neck, upper chest, and extremities [2, 3]. Nail abnormalities, predominantly affecting the fingernails more than toenails, may include nail thinning, longitudinal ridges, onychoschizia, and pterygium formation [4]. Beyond these hallmark findings, this telomeropathy is associated with progressive multiorgan involvement, particularly of the bone marrow, lungs, and liver [5, 6, 7]. While bone marrow failure remains the most frequently reported systemic complication, hepatic involvement is increasingly recognized but remains underreported and poorly characterized. The spectrum of liver disease ranges from mild hepatopathy and transaminitis to hypoplastic liver, hepatomegaly, portal hypertension, and even decompensated cirrhosis [5, 8]. In certain patients with telomere biology disorders, upper gastrointestinal bleeding due to esophageal varices may be the initial manifestation of advanced liver disease [9].

Here, we report two adult male patients with DC and decompensated CLD. This case series was prepared according to the CARE guidelines for standardized and transparent clinical reporting [10].

## 2 Case Report

### 2.1 Case 1

#### 2.1.1 Case History/Examination

A 21‐year‐old male presented to our outpatient department with a one‐month history of abdominal pain, localized to the left upper quadrant. The pain was dull, aching in character, associated with a dragging sensation, non‐radiating, and without any identifiable aggravating or relieving factors. He reported a significant episode of upper gastrointestinal (UGI) bleeding 4 years prior, during which UGI endoscopy and variceal band ligation were performed. He denied any history of alcohol consumption, hepatotoxic drug intake, or illicit drug use.

**FIGURE 1:**
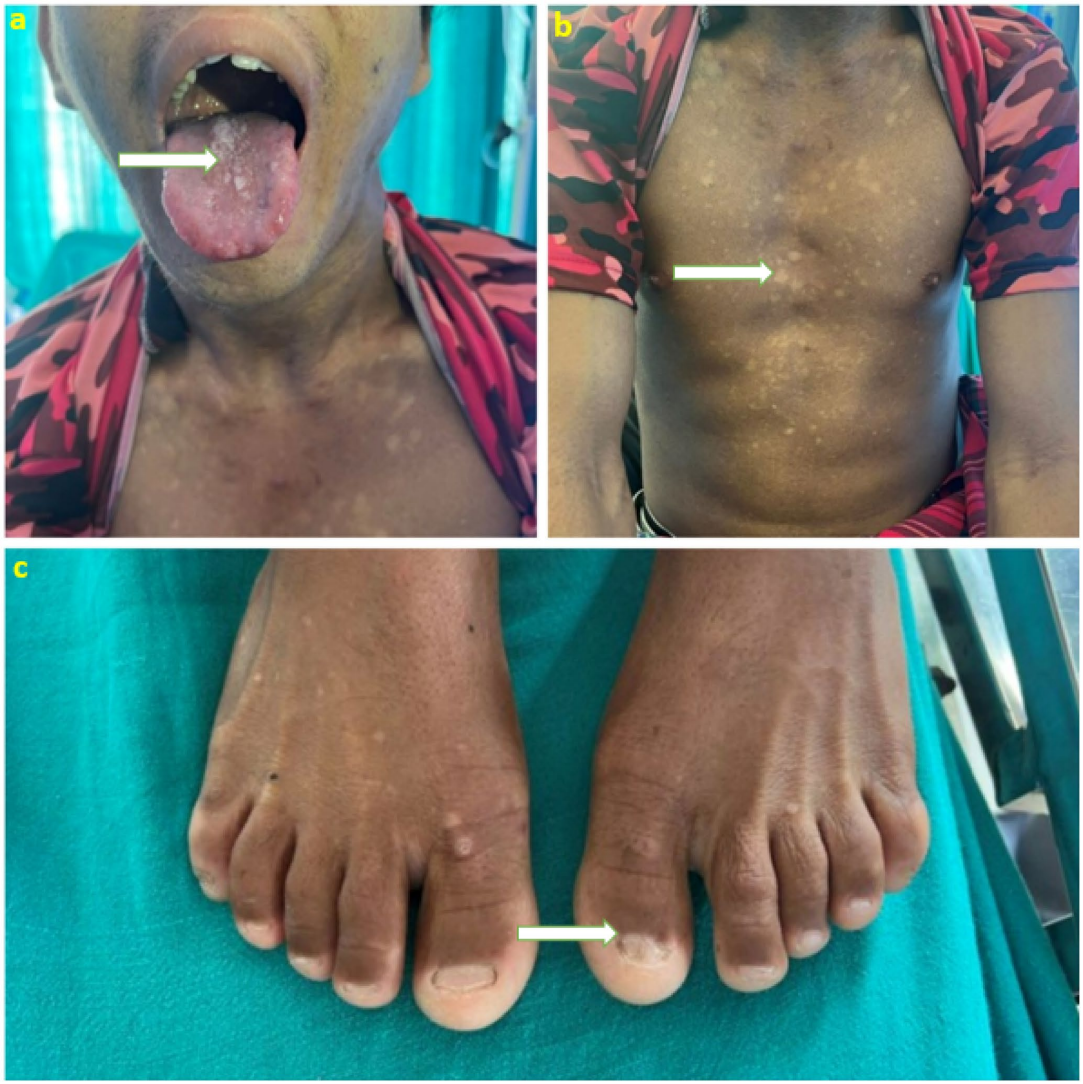
(a) Oral leukoplakia presenting as whitish plaques over the dorsum of the tongue—part of the diagnostic mucocutaneous triad of Dyskeratosis Congenita. (b) Reticular skin hyperpigmentation involving the upper limb, chest, and abdomen is consistent with the cutaneous manifestation of Dyskeratosis Congenita. (c) Nail dystrophy with longitudinal ridging and thinning of the fingernails, representing a classical feature of Dyskeratosis Congenita.

On general examination, the patient was well‐oriented to time, place, and person with no features suggestive of hepatic encephalopathy. Vital signs were within normal limits. Intraoral examination revealed well‐demarcated leukoplakic plaques over the dorsum of the tongue (Figure 1a). The patient had reticular hyperpigmentation of the skin (Figure 1b), dystrophic changes in the fingernails and toenails (Figure 1c), and a body mass index (BMI) of 21.5 kg/m^2^. Abdominal examination demonstrated a palpable spleen.

#### 2.1.2 Methods and Results

**FIGURE 2:**
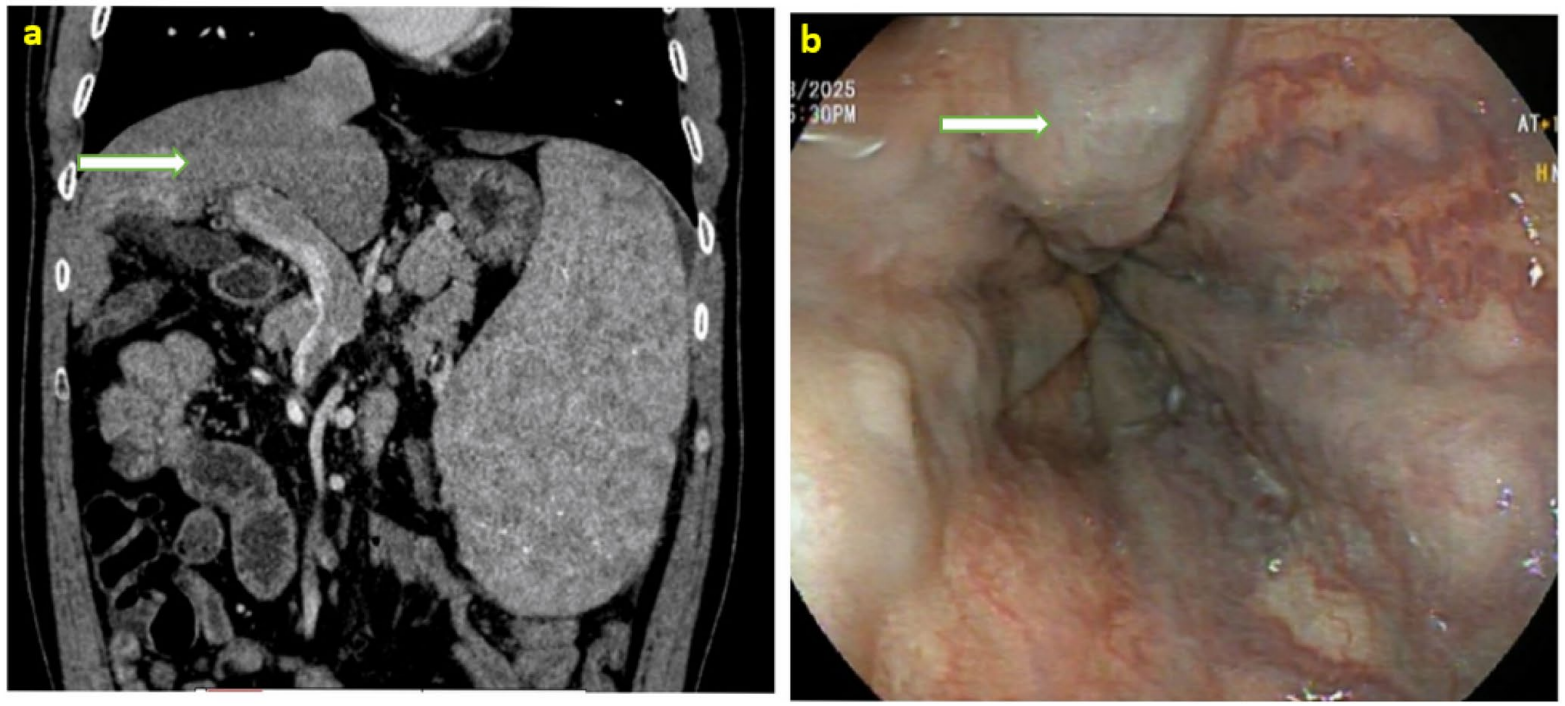
(a) CT abdomen (Arterial phase in coronal section with slice thickness of 1 mm) showing cirrhotic liver with dilated portal vein and splenomegaly. (b) Upper gastrointestinal endoscopy showing high‐risk esophageal varices (Grade III).

Initial laboratory investigations revealed bicytopenia, with a total leukocyte count of 3300/mm^3^ and a platelet count of 35,000/mm^3^, while hemoglobin was within normal limits. Liver function tests showed elevated total bilirubin (2.2 mg/dL, direct: 0.5 mg/dL), alanine transaminase (ALT) of 70 U/L, aspartate transaminase (AST) of 90 U/L, and albumin of 3.9 g/dL. Other relevant laboratory parameters showed normal serum creatinine (0.97 mg/dL), sodium (141 mmol/L), and normal prothrombin time (PT) of 16 s and INR (international normalized ratio) of 1.31. A triphasic computed tomography (TPCT) was performed, which showed changes of CLD with portal hypertension and splenomegaly with no liver lesion and ascites (Figure 2a). All the hepatic veins and inferior vena cava were patent both on ultrasound and CT scan. MELD‐Na score was 12 and CTP score was 6 (A‐grade).

Given the prior history of hematemesis, an upper gastrointestinal endoscopy was performed. The endoscopy revealed three columns of high‐risk esophageal varices (Figure 2b), mild portal hypertensive gastropathy (PHG). Fibroscan showed a liver stiffness measurement of 27.8 kPa. Endoscopic variceal band ligation (EVL) was performed. An extensive etiological work‐up for chronic liver disease was initiated. Viral serologies for hepatitis B and C were non‐reactive. Other etiological workups, including for Wilson's disease and autoimmune hepatitis, were noncontributory. Fasting blood glucose, lipid profile, and serum iron indices were within normal limits, as shown in Table 1. There was no significant history of drug or toxin exposure. Liver biopsy was not done due to a lack of consent. Given the presence of characteristic mucocutaneous features, including reticular skin pigmentation, oral leukoplakia, and nail dystrophy, a dermatologic consultation was obtained, and a clinical diagnosis of dyskeratosis congenita was made. Genetic testing and skin biopsy were advised for the patient. However, the patient did not provide consent for these tests because of financial constraints. Since other etiological workups were inconclusive, we considered DC as the most likely cause of CLD.

**TABLE 1:** Table showing relevant investigations.

Relevant lab details	Case 1	Case 2	Normal lab range	Method of analysis
Hemoglobin (gram/dL)	13.2	5.7	12–16	Cyanmethemoglobin
Total leukocyte count (per mm^3^)	3300	930	4000–11,000	Impedance
Platelet count (per mm^3^)	35,000	28,000	1,50,000–4,00,000	Impedance
Total bilirubin (mg/dL)	2.2	0.7	0.4–1.2	Jendrassik and Grof/Diazonium Salt
Direct bilirubin (mg/dL)	0.5	0.2	0.1–0.4	Jendrassik and Grof/Diazonium Salt
Alanine Transaminase (Unit/L)	70	32	< 45	UV kinetic with PLP (P‐5‐P)
Aspartate transaminase (Unit/L)	90	22	< 40	UV kinetic with PLP (P‐5‐P)
Albumin (gram/dL)	3.9	2.3	3.4–5.5	Bromocresol Purple (BCP)
Sodium (mmol/L)	141	137	135–150	Indirect ISE (Ion Selective Electrode)
Creatinine (mg/dL)	0.97	1.1	0.4–1.4	Jaffe's Kinetic/Alkaline Picrate
Prothrombin Time (PT in seconds)	16	14	10–14	Photo‐Optical Clot Detection (Standard prothrombin time of 13 s)
INR	1.31	1.10	0.8–1.1	Calculated (ISI‐1.31)
HCV IgM	Non‐reactive	Non‐reactive		ELISA
HBsAg	Non‐reactive	Non‐reactive		ELISA
HIV IgM	Non‐reactive	Non‐reactive		ELISA
AMA, ANA, ASMA titer	Negative	Negative	< 1:40	Indirect IFA
FBS (mg/dL)	96	101	70–100	Hexokinase
Ferritin level (ng/mL)	35	23	10–291	CLIA
Serum iron (mcg/dL)	62	101	50–175	Direct Method (Ferene)
Total iron binding capacity (mcg/dL)	310	267	250–450	Colorimetry
24‐h urine copper (mcg/24 h)	15	6	10–30	FAAS (Flame Atomic Absorption Spectrophotometry)
Serum ceruloplasmin level (mg/dL)	31	25	22–40 (for male)	Immunonephelometry
Alpha‐1 Antitrypsin Deficiency	Not available	Not available	—	—
Genetic testing	Not available	Heterozygous missense mutation in NOP 10 gene (p.Tyr6Cys c.17A>G variant)		Next Generation Sequencing (NGS)
Bone marrow biopsy	Not available	Hypocellular marrow with reduced trilineage hematopoiesis		
UGI endoscopic findings	High‐risk esophageal varices	High‐risk esophageal varices with signs of recent hemorrhage		Fujinon's gastroscope (EG 760R)
Triple phase CT abdomen	CLD changes with features of portal hypertension and splenomegaly	CLD changes with features of portal hypertension and splenomegaly		
USG Doppler of the splenoportal axis	No evidence of portal vein obstruction and hepatic venous outflow tract obstruction	No evidence of portal vein obstruction and hepatic venous outflow tract obstruction		

Abbreviations: AMA, anti‐mitochondrial antibody; ANA, anti‐nuclear antibody; ASMA, anti‐smooth muscle antibody; CLIA, chemi‐luminescence immuno‐assay; CT, computed tomography; ELISA, Enzyme Linked Immunosorbent Assay; FBS, fasting blood sugar; HBsAg, hepatitis B surface antigen; HCV, hepatitis C virus; HIV, human immunodeficiency virus; IFA, immuno‐fluorescence assay; UGI, upper gastrointestinal; USG, ultrasonography; UV kinetics with PLP (P‐5‐P), Ultraviolet kinetics with Pyridoxal‐5‐phosphate.

### 2.2 Case 2

#### 2.2.1 Case History/Examination

**FIGURE 3:**
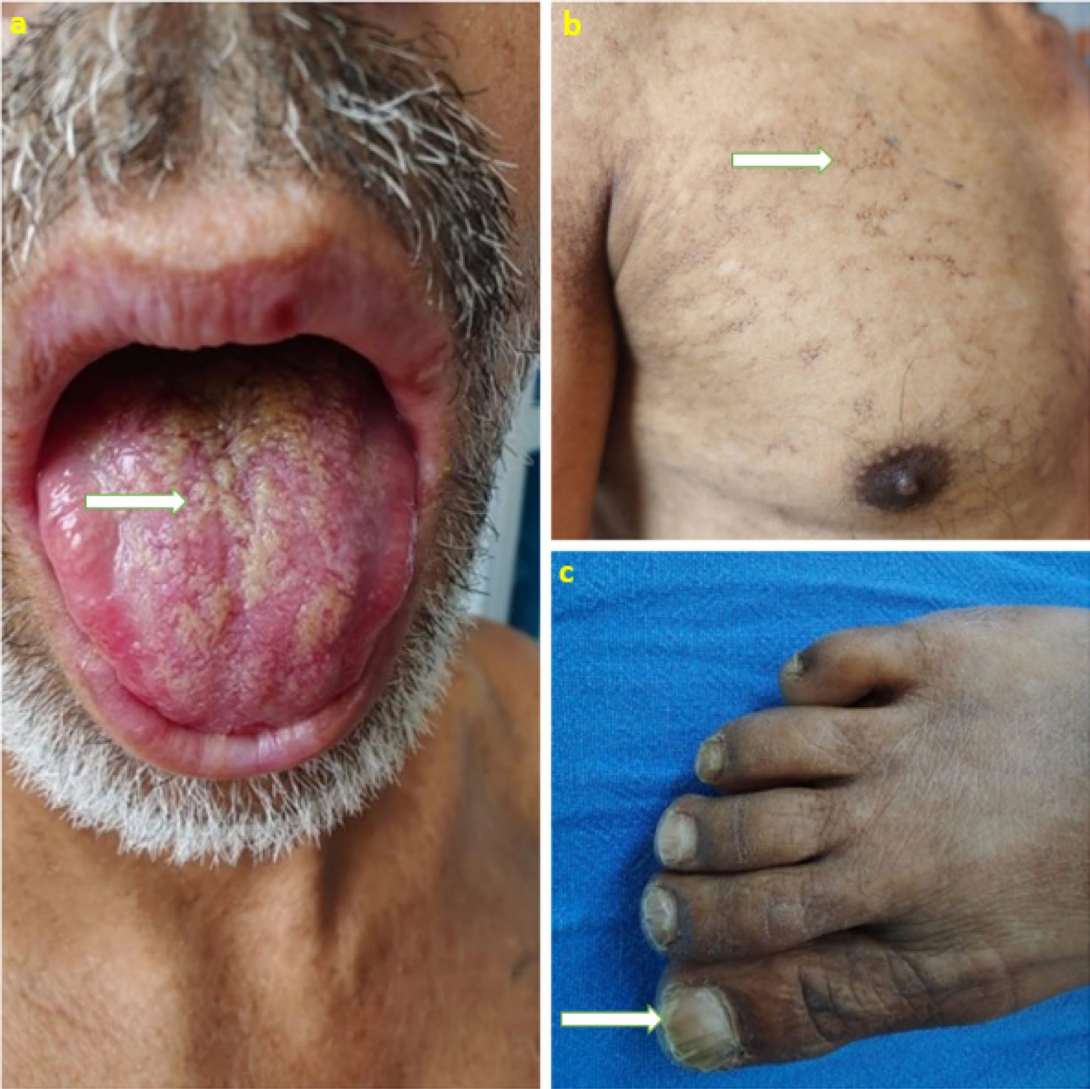
(a) Oral leukoplakia presenting as whitish plaques over the dorsum of the tongue—part of the diagnostic mucocutaneous triad of Dyskeratosis Congenita. (b) Reticular skin hyperpigmentation involving the upper limb and chest, consistent with the cutaneous manifestation of Dyskeratosis Congenita. (c) Nail dystrophy with longitudinal ridging and thinning of the fingernails, representing a classical feature of Dyskeratosis Congenita.

A 35‐year‐old male patient presented with a history of recurrent upper gastrointestinal (UGI) bleeding and episodes of intermittent fever over the past 6 months. He presented with the third episode of UGI bleeding. After initial resuscitation, UGI endoscopy was performed, which showed high‐risk esophageal varices with signs of recent hemorrhage. Endoscopic variceal ligation was performed. The patient was oriented well to time, place, and person. There was no flapping tremor on examination. On head‐to‐toe examination, the patient had oral leukoplakia (Figure 3a), distinct reticular skin hyperpigmentation (Figure 3b), and typical dystrophic nail changes (Figure 3c). A dermatological opinion was obtained, and a clinical diagnosis of DC was established.

#### 2.2.2 Methods and Results

All the documents from previous hospitals were assessed, which revealed that the patient had been evaluated in the past for pancytopenia. A bone marrow biopsy report revealed hypocellular marrow with reduced trilineage hematopoiesis. Fanconi anemia was excluded by chromosomal breakage analysis. Next‐generation sequencing (NGS) identified a heterozygous missense mutation (p.Tyr6Cys c.17A>G variant) in the NOP10 gene, consistent with a diagnosis of Dyskeratosis congenita (DC). Blood workup performed at our center showed pancytopenia: hemoglobin of 5.7 g/dL, platelet count of 28,000/mm^3^, and total leukocyte count of 930/mm^3^ with an absolute neutrophil count (ANC) of 613/mm^3^. Liver function tests revealed hypoalbuminemia (2.3 g/dL), while bilirubin and transaminase levels were within normal limits. Other laboratory parameters showed normal renal function with serum creatinine (1.1 mg/dL), sodium (137 mmol/L), and normal prothrombin time (PT) of 14 s and INR (international normalized ratio) of 1.10. MELD‐Na score was 8 and CTP score was 7 (B–grade). A TPCT abdomen was performed, which showed features of CLD, portal hypertension, and a massive spleen without evidence of ascites. There was no hepatic lesion, and the hepatic veins and inferior vena cava were patent. Liver stiffness measurement was 25.2 kPa, indicating stage 4 fibrosis. Liver biopsy was not done due to a lack of consent. A positive family history was noted—two of his brothers, who had similar reticular skin pigmentation, died from complications of hematological malignancies.

General examination revealed the presence of florid oral leukoplakia, as shown in Figure 3a. As depicted in Figure 3b, dermatologic examination revealed diffuse, lace‐like reticular hyperpigmentation distributed over the forearm, chest, abdomen, and back. Additional mucocutaneous findings included dystrophic nail changes, as shown in Figure 3c. The patient also exhibited premature graying of hair and patchy alopecia, consistent with other features of dyskeratosis congenita.

Other etiological workups for viral hepatitis, autoimmune hepatitis, metabolic dysfunction‐associated liver disease, and Wilson's disease were not contributory, as shown in Table 1. The patient never consumed alcohol and had no other known prior comorbidities.

### 2.3 Follow‐Up

A relook UGI endoscopy was performed in the second case after 29 days of the first endoscopy, which showed small, low‐risk residual varices. Pulmonology consultation was also obtained on an OPD basis for any needful, but both patients were advised for conservative management after normal pulmonary function tests. Both patients are taking beta blocker (carvedilol) in optimal dosage on a daily basis. Lifestyle modifications have been explained properly, including salt restriction, adequate nutritional intake, and other possible risk factors of disease progression, like sunlight exposure, tobacco smoking, and potential occupational hazards. Both patients have been explained about the course and prognosis of the disease, including risks of acute hepatic decompensation, and counseled regarding the potential benefit of liver and bone marrow transplantation in higher centers of convenience. However, both opted for conservative management owing to financial constraints. Both patients are under follow‐up on a regular basis (2–3 monthly) with regular monitoring of liver and renal function tests and hemogram. Variceal surveillance has been planned annually.

## 3 Discussion

Dyskeratosis congenita (DC) is a rare inherited disorder of telomere biology, characterized by a classical mucocutaneous triad of reticular skin hyperpigmentation, nail dystrophy, and oral leukoplakia [1]. In addition to these hallmark features, DC frequently involves multiple organ systems, including the bone marrow, lungs, and liver, reflecting its systemic nature [6, 7]. In this case series, we reported two male patients with DC presenting with advanced liver disease and complications of portal hypertension.

Hepatic involvement in DC is rare, occurring in 7%–10% of patients with DC. However, DC is increasingly being recognized as a cause of liver disease. The pathogenesis of liver disease in Dyskeratosis Congenita remains incompletely understood. However, emerging evidence suggests that telomere shortening in hepatic stellate cells and vascular endothelial cells may impair hepatic regeneration, promote fibrosis, and contribute to the development of portal hypertension [11, 12, 13]. The spectrum of hepatic involvement in DC ranges from mild transaminitis and hepatomegaly to progressive fibrosis, portal hypertension, cirrhosis, and its complications [11, 14, 15, 16, 17]. In a longitudinal cohort study by Vittal et al., 72% of patients with dyskeratosis congenita or related telomere biology disorders had abnormal liver enzymes at baseline, and 17.2% developed clinically significant liver disease with portal hypertension over a median follow‐up period of 6 years [11]. Both patients in the current series presented in a state of hepatic decompensation in the form of variceal bleeding. Though histological diagnosis was not attempted, the clinical features, endoscopic findings, cross‐sectional imaging, and liver stiffness measurements were diagnostic of chronic liver disease with clinically significant portal hypertension. Cirrhosis in diseases of telomere activity, such as DC, is commonly associated with mutations in TERT and TERC. However, one of our cases had a rare genetic mutation involving the NOP10 gene, which is a new finding.

Bone marrow failure is a hallmark of DC, and almost all patients eventually develop cytopenias [18, 19]. By age 30, approximately 80%–90% of individuals show significant marrow failure, often manifesting as pancytopenia with associated risks of opportunistic infections and bleeding [20]. While pancytopenia can be due to portal hypertension in DC patients with liver disease, a bone marrow study in one of our patients was consistent with marrow involvement. The same patient had recurrent episodes of fever and hospitalization and a critically low absolute neutrophil count, indicating that these patients are at high risk of complications of marrow suppression. Lung involvement was not observed in either patient. Though patients with DC are at heightened risk of malignancy, no evidence of malignancy was seen in either of our patients.

An extensive workup was done to rule out different etiologies of CLD. This is important because treating the etiology can reverse or slow the progression of CLD in some cases. Despite a thorough workup, rare causes of CLD remain underinvestigated. For instance, treatable infectious causes like visceral leishmaniasis, which presents with cytopenia and fever with acute liver dysfunction and features of secondary HLH, are common in this part of the world and should always be considered as one of the possible treatable causes [21].

Management of chronic liver disease and portal hypertension in DC is the same as the standard of care for CLD patients. However, concomitant marrow and pulmonary involvement can complicate the scenario, requiring multidisciplinary assistance in these patients. We performed variceal band ligation for bleeding in both patients. Dyskeratosis congenita and associated CLD are both progressive diseases and eventually result in end‐stage conditions necessitating marrow and liver transplantation for a better long‐term prognosis. Bone marrow transplantation for severe marrow failure followed by liver transplantation for decompensated CLD has been found to be a successful treatment option in these patients [5, 7]. Both patients were counseled about the need for liver and bone marrow transplantation in the days to come.

Case reports have their own limitations. It is important to acknowledge that genetic testing to establish the diagnosis of DC was not performed in one of the patients, given financial constraints. Though the sensitivity of clinical features in diagnosing DC has low sensitivity, the presence of the classic mucocutaneous triad is almost diagnostic in the absence of genetic testing. Moreover, genetic testing may turn out positive only in 50%–70% of cases due to mutations in unknown genes [22]. In addition, all other usual etiologies of CLD were ruled out before establishing the diagnosis of DC in the first case.

## 4 Conclusion

Chronic liver disease can result from various etiologies. Dyskeratosis congenita is a rare telomeropathy increasingly recognized to be associated with CLD. In cases of cryptogenic CLD where no obvious cause can be demonstrated, active screening for the characteristic mucocutaneous triad of dyskeratosis should be considered. The presence of the classic mucocutaneous triad almost establishes the diagnosis of DC. Though DC largely remains untreatable, an early diagnosis of CLD in these patients is of paramount importance since it provides an opportunity to anticipate and treat complications of CLD timely. Our case report emphasizes the association between telomeropathy, like DC and CLD.

## Author Contributions

**Bigyan Maharjan:** data curation, methodology, software, writing – original draft, writing – review and editing. **Pradeep Neupane:** data curation, methodology, software, writing – original draft, writing – review and editing. **Bikash Poudel:** data curation, methodology, software, writing – original draft, writing – review and editing. **Ramesh Jha:** data curation, methodology, software, writing – original draft, writing – review and editing. **Suraj Subedi:** data curation, methodology, software, writing – original draft, writing – review and editing. **Jessica Baral:** data curation, methodology, software, writing – original draft, writing – review and editing. **Abhishek Pandit:** data curation, methodology, software, writing – original draft, writing – review and editing. **Shraddha Uprety:** data curation, methodology, software, writing – original draft, writing – review and editing. **Mukesh Kumar Ranjan:** conceptualization, data curation, investigation, methodology, project administration, software, supervision, validation, visualization, writing – original draft, writing – review and editing.

## Funding

The authors have nothing to report.

## Consent

Patient allowed personal data processing, of and informed consent was obtained from the patient and uploaded.

## Conflicts of Interest

The authors declare no conflicts of interest.

## Data Availability

The authors have nothing to report.
